# Antimalarial Activity of Crude Extract and Solvent Fractions of the Leaves of *Bersama abyssinica* Fresen. (Melianthaceae) against *Plasmodium berghei* Infection in *Swiss* Albino Mice

**DOI:** 10.1155/2020/9467359

**Published:** 2020-06-09

**Authors:** Agumas Alemu Alehegn, Jibril Seid Yesuf, Eshetie Melese Birru

**Affiliations:** ^1^Department of Pharmacy, Lumame Primary Hospital, Lumame, Ethiopia; ^2^Department of Pharmacology, School of Pharmacy, College of Medicine and Health Sciences, University of Gondar, Gondar, Ethiopia

## Abstract

**Background:**

Treatment of malaria has been compromised by the emergence of drug-resistant parasites. Consequently, novel agents are urgently needed from different sources including from medicinal plants. Thus, the current study aimed at evaluating the antimalarial activity of crude extract and solvent fractions of the leaves of *Bersama abyssinica (B. abyssinica)* against *Plasmodium berghei* infection in *Swiss* Albino mice.

**Method:**

A 4-day suppressive test was employed to evaluate the antimalarial effect of crude extract and solvent fractions against early infection. The curative and prophylactic effects of crude extract and fraction with the highest chemosuppression were further tested by Rane's test and residual infection procedure. Parasitemia, survival time, packed cell volume (PCV), body weight, and rectal temperature of mice were used as evaluation parameters. Windows SPSS version 20 was used to analyze the data and analysis of variance (ANOVA) followed by Tukey's post hoc test was used to compare data between groups.

**Results:**

The crude extract and aqueous fraction significantly (*P* < 0.05 to 0.001) suppressed parasitemia followed by protection of PCV reduction resulting in prolonging the survival time but failed to protect body weight and rectal temperature reduction in all tested models. The ethyl acetate and chloroform fractions also showed significant chemosuppression and PCV protection in the 4-day suppressive test. The crude extract exhibited a chemosuppression of 49.51%, 57.94%, and 44.11% while the aqueous fraction showed suppression of 47.69%, 51.62%, and 37.07% in 4-day suppressive, curative, and prophylactic tests, respectively, at 400 mg/kg.

**Conclusion:**

The crude extract and fractions showed fairly moderate antimalarial activity, and the finding supports the traditional claims and previous *in vitro* studies. Thus, this may call for further studies to isolate chemical entities for additional safety and efficacy tests.

## 1. Background

Malaria is the most prevalent and devastating mosquito-borne infectious disease. It is a major cause of morbidity and mortality throughout tropical and subtropical regions of the world, where the temperature and rainfall are suitable for the development of vectors and parasites [1]. An estimated 228 million cases and 405,000 deaths of malaria occurred worldwide in 2018. Almost 80% of all malaria cases and deaths globally were in the African region and in India [2]. The burden of disease can be exceptionally high among the most vulnerable groups, such as children <5 years old, pregnant women, and migrant laborers traveling to endemic areas, especially when worsening nutritional conditions impair their capacity to fight the disease [3].

Malaria is also the major public health problem in Ethiopia and it is prevalent in over 75% of the country's area. Approximately 68% of the Ethiopian population have been estimated to live in areas of <2,000 m of altitude and, thus, are considered to be at risk of malaria [2, 4]. *P. falciparum* and *P. vivax* are the most dominant malaria parasites in Ethiopia and they are prevalent in all malarious areas in the country accounting for 60–70% and 30–40% of malaria cases, respectively, although their relative composition can be variable. *P. malariae* and *P. ovale* are rare and account for <1% of all confirmed malaria cases [5].

Due to the prevalence of malaria as well as the growing incidence of deaths coupled with the alarming rate of resistance to antimalarial drugs and pesticides, restriction in the use of chemical sprays, adverse reactions of antimalarial drugs, inaccessibility, the problem of affordability, the drying out of new antimalarial drug pipeline, lack of an effective vaccine, and the limited number of effective antimalarial drugs, discovering new antimalarial compound is more than ever a priority [6]. So, new antimalarial agents that are safe, more effective, and affordable with broad therapeutic potential, structurally distinct from existing drugs, and being readily available with a novel mode of action are urgently needed [7]. These have stimulated the search for new pharmacologically active agents that can overcome these barriers from natural products, because history reveals that both quinine [8] and artemisinin [9] have been derived from traditional medicine and plant extracts.

The studied plant*, B. abyssinica* Fresen. (Melianthaceae), is a species of small- or medium-sized evergreen shrub to a small tree of 6–9 m, rarely exceeding 25 m in height. In Ethiopian traditional medical practices, where it is commonly identified as azamirr, the dried and powdered leaves of the plant [10], decoction of stem barks, leaves and roots [11, 12], and homogenization of the leaves [13] have been reported for the management of malaria. The decoction of the stem bark, leaves, and roots in Tanzania [14], chewing leaves and seeds in Kenya [15], and the leaf parts in central west Ivory Coast [16] have been also reported for malaria management. The stem bark extract has been reported to have substantial in vitro antiplasmodial activity with 86.67 ± 11.32% of inhibition rate against chloroquine-resistant *P. falciparum* Dd2 malaria parasite strain at 100 *µ*g/ml of the 80% ethanol crude extract [14] and good antimalarial activity against *P. falciparum* FCA-20/Eth strain with IC50 of 11 *µ*g/ml [17]. On the other hand, ethanol extract of the leaves has been reported to have moderate *in vitro* antiplasmodial activity (IC50 = 23.9 ± 5.7 *µ*g/ml) against chloroquine-resistant FcB1/Colombia strain of *P. falciparum* [16] and pronounced antimalarial activity against chloroquine-sensitive strain (FCA-20/Eth) (IC50 = 4 *µ*g/ml) [17]. Thus, based on ethnobotanical use and *in vitro* studies mentioned above, the present study evaluated the *in vivo* antimalarial activity of the crude extract and solvent fractions of the leaves of *B. abyssinica.*

## 2. Materials and Methods

### 2.1. Collection of Plant Materials

The leaves of *B. abyssinica* were collected from Tara Gedam, South Gondar Zone, Amhara National Regional State, 85 km away from Gondar city, in December 2018. The collected plant material was wrapped with plastic sheets during transportation, authentication was done by a botanist in the Biology Department, University of Gondar, and a voucher specimen (voucher no. 001AAA) was deposited in the herbarium of Department of Biology, University of Gondar, for future reference.

### 2.2. Experimental Animals and Parasites

A total of 170 healthy, adult Swiss Albino mice of either sex (25 ± 5 g, 6 to 8 weeks of age) were purchased from the Ethiopian Public Health Institute (EPHI). The animals were kept in cages and housed in a standard animal house under a natural 12/12 h light-dark cycle at room temperature and provided with pellet diet and water *ad libitum* in the animal house of the Department of Pharmacology, School of Pharmacy, College of Medicine and Health Sciences, University of Gondar. The mice were maintained and cared as per the international guidelines for the use and maintenance of experimental animals throughout the experiment [18, 19]. Animals were allowed to acclimatize to the laboratory condition for a week before the beginning of the experiment.

The *Plasmodium berghei* ANKA strain (chloroquine-sensitive) was obtained from EPHI, Addis Ababa, and the parasite was maintained by intraperitoneal serial passage of blood from mouse to mouse.

### 2.3. Preparation of Plant Material

The collected plant leaves were cleaned with tap water and air-dried at room temperature under a shade and reduced to appropriate size. Then, it was packed in a plastic bag and kept until extraction. The powdered plant materials were weighed by sensitive digital weighing balance (EPH-400 Abron Exports), and a total of 1000 grams of powdered leaves was macerated with 80% methanol (250 g in 1500 ml) in Erlenmeyer flask for 72 hours at room temperature. The extraction process was facilitated by occasional shaking. After 72 hours, the extract was separated from the marc using gauze and further filtered by Whatman filter paper No. 1. The residue was remacerated for another 72 hours two times using the same volume of 80% methanol. After exhaustive extraction, the combined filtrate was dried by hot air oven (Medit-Medizin Technik, Germany) at 40°C to obtain the crude extract of the plant. The extract was further concentrated to dryness by freeze-drying using Lyophilizer (LabFreez Instruments, Germany). After drying, the amount of dry extract obtained was harvested and the dried extract was transferred into vials and kept in a desiccator at −4°C until use. The weight of the dry extract was expressed as a percentage of the total mass of dry plant matter to determine the percentage yield [20]. About 80 grams of the crude extract was used for fractionation and the remaining was used for the antimalarial activity evaluation of the crude extract.

### 2.4. Fractionation

The dried crude 80% methanolic leaf extract of *B. abyssinica* was further fractionated using sequential solvent partitioning by different solvents of increasing polarity (chloroform, ethyl acetate, and water) to get different solvent fractions. Eighty grams of the extract was suspended in 400 ml of distilled water in a separatory funnel. The aqueous portion was partitioned three times with 400 ml of chloroform to obtain chloroform fraction. Then, the aqueous residue was further fractionated three times with 400 ml of ethyl acetate to obtain ethyl acetate fraction. In the end, from the remaining aqueous solution, the third fraction, which we call aqueous fraction, was obtained. The first two fractions were dried in an oven under 40°C, while the aqueous fraction was solidified in a refrigerator overnight and then dried using a Lyophilizer. The % yield of the dried fractions was calculated, and the fractions obtained were put in airtight bottles and stored in a refrigerator at −4°C until used [21].

### 2.5. Acute Oral Toxicity Testing

Acute oral toxicity of the crude extract was performed on healthy female Swiss Albino mice as per the OECD-425 guideline limit test procedure [22]. Accordingly, five female albino mice of 6–8 weeks of age were used. All mice were fasted (food but not water) for 3 hours before and 1 hour after the administration of the extract. A limit dose of 2000 mg/kg was given for the first animal. Based on the outcome of the first mouse, the crude extract was administered for four additional mice. The mice were housed separately and observed for the manifestation of gross behavioral and physical toxicities like changes in the skin, urination, lacrimation, reduction in feeding activity, excitation, paw licking, increased respiratory rate, decreased motor activity, diarrhea, weight loss, and paralysis continuously for the first 30 minutes and intermittently for 4 hours, over a period of 24 hours and later followed for 14 days for an interval of 24 hours for any lethality.

### 2.6. Grouping and Dosing of Animals

Mice were randomly assigned into three extract-treated groups and two controls, six mice per group for each. Group I was a negative control and received distilled water which was used as a solvent for both the crude and solvent fraction extracts. Groups II, III, and IV were treated with 100, 200, and 400 mg/kg doses of the extract orally, respectively. The doses of the extract were 1/20^th^, 1/10^th^, and 1/5^th^ of the LD50 value from the acute oral toxicity study. Group V was treated with chloroquine 25 mg/kg via the oral route using oral gavage. The volume administered was calculated based on individual mouse body weight and duration of administration depended on the type of test performed.

### 2.7. Inoculation of Parasite

Mice were infected by a blood sample collected from a donor mouse with a rising parasitemia of about 20–30%. After the determination of the percentage parasitemia in the donor mouse, it was sacrificed by head blow and blood was collected via incisions of the jugular vein into a test tube containing 3.8% trisodium citrate added as an anticoagulant. The collected blood was diluted by 0.9% physiological isotonic saline based on the parasitemia of the donor mice and the RBC count of normal mice (4.5 × 10^9^ RBC/ml) in such a way that 1 ml blood contains 5 × 10^7^*P. berghei*-infected erythrocytes. Each mouse to be used in the experiment was injected intraperitoneally with 0.2 ml of infected blood containing about 1 × 10^7^*P. berghei*-parasitized erythrocytes [23].

### 2.8. Four-Day Suppressive Test

This test was conducted to evaluate the schizonticidal activities of the plant material on early *P. berghei* infection in mice as described by Knight and Peters [24]. The infected mice were randomly divided into five groups, and treatment was started three hours after the inoculation of the parasite on day 0 and continued for the next four days from day 0 to day 3 with a 24-hour time interval between the doses. After giving the treatment for four days, thin blood film was made from the tail of each mouse on the fifth day (D4) to determine the level of parasitemia and percentage inhibition.

### 2.9. Rane's Test (Curative Test)

The curative test was undertaken with the crude extract and aqueous fraction, which showed the highest parasitemia suppression in the 4-day suppressive test. Accordingly, evaluation of the curative potential against an established malaria infection was carried out according to the method described by Ryley and Peters [25]. The mice were injected intraperitoneally with a standard inoculum of 1 × 10^7^*P. berghei*-infected erythrocytes on the first day (day 0). Seventy-two hours later, the mice were divided into five groups of six mice per group for both the crude extract and solvent fractions. The mice were treated once daily for 5 days from day 3 to day 7. Starting from day 3 through day 7 daily, thin blood films were prepared from the tail bleed of each mouse to monitor the level of parasitemia.

### 2.10. Test for Prophylactic Activity

The prophylactic activity of the crude extract and the aqueous fraction, which showed the highest parasitemia suppression in the 4-day suppressive test, was tested using the residual infection procedure described by Peters [26]. Groups of mice were randomized into five groups of six mice per group for crude extract and aqueous fraction. Treatment was given daily for four days, and all mice were infected with the parasite on the 5th day. Thin blood films were prepared from each mouse after 72 hours of infection to determine the level of parasitemia.

### 2.11. Determination of Parasitemia

Thin blood smears were prepared from the tail snip of each mouse on the fifth day (D4) for Peter's 4-day suppressive test, after 72 hours of infection on day 8 in the prophylactic test and from day 3 after infection established to day 7 for the curative test on microscopic slides. The slides were dried, fixed with absolute methanol, and stained with 10% Giemsa at pH 7.2 for 10 minutes, and then, they were washed gently using distilled water and air-dried at room temperature. Finally, the slides were examined under a microscope with an oil immersion objective (×100 magnification power) by taking an average of six fields. The parasite count was done by an experiment-blinded technician. Percentage parasitemia was calculated by counting infected RBC and total RBC from Giemsa-stained thin blood films, and the average percentage suppression of parasitemia was calculated for each dose level by comparing the parasitemia in infected controls with that of treated mice with the following formulas [27]:

(1) $$\begin{array}{c} \%\text{ parasitemia}=\frac{\text{number of parasitized RBCs }}{\text{total number of RBCs}}\;*\;100\%,\\ \%\text{ suppression}=\frac{\left(\text{mean parasitemia of negative control}-\text{mean parasitemia of the treated group}\right)}{\text{mean parasitemia of negative contol }}\;*\;100\%. \end{array}$$

### 2.12. Determination of Mean Survival Time

Mortality was monitored daily and the number of days from the time of inoculation of the parasite up to death was recorded for each mouse in the treatment and control groups throughout the follow-up period of 30 days (D0–D29) for all the models. Mean survival time (MST) for each group was determined arithmetically by calculating the average survival time (days) of mice starting from the date of infection over a period of 30 days (D0–D29). MST for each group was then calculated using the following formula [28]:

(2) $$\begin{array}{c} \text{MST}=\frac{\text{sum of survival time for all mice in a group}\left(\text{in days}\right)}{\text{total numbers of mice in that group}}. \end{array}$$

### 2.13. Packed Cell Volume Determination

Packed cell volume (PCV) is the other antimalarial activity evaluation parameter to assess whether the test substance is effective in preventing hemolysis associated with the invading *Plasmodium* parasite or not. Blood was collected from the tail of each mouse in heparinized microhematocrit capillary tubes. The capillary tubes were filled to 3/4^th^ of their height with blood and sealed at one end with sealing clay. The tubes were then placed in a microhematocrit centrifuge, with the sealed end being outwards and centrifuged for 5 minutes at 11,000 rpm. The tubes were taken out of the centrifuge, and PCV was determined using a standard Micro-Hematocrit Reader. It was measured before inoculating the parasite (day 0) and day 4 in Peter's 4-day suppressive test and D0 and D7 in the prophylactic activity test. In the case of Rane's test, PCV was measured on the third day after infection established and on the last day of treatment on the seventh day. PCV is a measure of the proportion of RBCs to plasma in the whole blood and is determined using the relation shown as follows [29]:

(3) $$\begin{array}{c} \text{PCV}=\frac{\text{volume of erythrocytes in a given volume of blood}}{\text{total blood volume }}. \end{array}$$

### 2.14. Determination of Body Weight and Temperature Change

The body weight of each mouse in all groups was measured on day 0 before infection and day 4 in the four-day suppressive test and D0 and D7 (72 hrs after infection) in the prophylactic activity test while in Rane's test it was measured on day 3 after the infection was established and on day seven, the last day of the treatment, using a sensitive digital weighing balance, to observe whether the test extract of the leave prevents body weight loss that commonly reduced with increasing parasitemia in infected mice. The rectal temperature of each mouse in all groups was measured by a digital thermometer one hour before infection, four hours after infection, and then daily to see the effect of crude extract and solvent fractions on the rectal temperature in the 4-day suppressive test. On the other hand, in Rane's test, rectal temperature was measured one hour before infection and then daily from day 3 to day 7, and in the prophylactic activity test, rectal temperature was measured on D0 before treatment, D4 before inoculation, and then daily till day 7.

### 2.15. Data Management and Statistical Analysis

The results were calculated by Microsoft Excel 2013 and expressed as mean ± SEM for each group. All the grouped data were statistically evaluated, and the significance of various treatments was calculated using one-way ANOVA followed by Tukey's post hoc test. The results were considered statistically significant at a 95% confidence level and *P* value < 0.05. All data processing was done using SPSS data analysis software version 20.

## 3. Results

### 3.1. Yield of Crude Extract and Fractions

As summarized in Table 1, from 120 grams of 80% methanol crude extract, the aqueous fraction provided the highest yield followed by the chloroform fraction, while the lower yield was obtained from the ethyl acetate fraction.

### 3.2. Acute Toxicity Test of Crude Extract

The acute toxicity study indicated that the crude extract caused no mortality in a limited dose of 2000 mg/kg within the first 24 hours as well as for the following 14 follow-up days. Physical and behavioral observations of the experimental mice also revealed no visible signs of overt toxicity. This indicates that LD50 of the extract is greater than 2000 mg/kg.

### 3.3. Effect of Crude Extract and Solvent Fractions in the 4-Day Suppressive Test

As presented in Table 2, the 80% methanolic crude extract and all solvent fractions of the leaves of *B. abyssinica* showed statistically significant (*P* <  0.05 to *P* < 0.001) chemosuppressive activity against *P. berghei* infection in mice as compared to the negative control. The highest level of inhibition (49.51%, *P* < 0.001) was obtained by 400 mg/kg dose of the crude extract, followed by the aqueous fraction (47.69%, *P* < 0.001) at 400 mg/kg, while the lowest suppression (28.05%, *P* <  0.05) was obtained after the administration of 100 mg/kg dose of the chloroform fraction. The rank order of chemosuppression of the solvent fractions was aqueous (47.69%, *P* < 0.001) > ethyl acetate (41.89%, *P* < 0.01) > chloroform (38.21%, *P* < 0.01) at 400 mg/kg dose. Comparison among the crude extract- and solvent fraction-treated groups did not show any significant differences. Chemosuppressive activities produced by all doses of crude extract and fractions were significantly (*P* < 0.001) lower than the standard drug, chloroquine 25 mg/kg, which showed 100% chemosuppression.

The crude extract at 200 and 400 mg/kg doses prolonged MST of mice significantly (10.50± 0.76 days and 13.83 ± 1.05 days, respectively, *P* < 0.001) as compared to the negative control (6.17 ± 0.40). The aqueous fraction at doses of 200 (10.5 0 ± 0.50 days, *P* < 0.05) and 400 mg/kg (13.67 ± 1.05 days *P* < 0.001) as well as the ethyl acetate fraction at a dose of 400 mg/kg (12.00 ± 1.77 days, *P* < 0.05) also showed statistically significant prolongation of mean survival time when compared with the negative control group (6.33 ± 0.62). Comparison among the crude extract-treated groups demonstrated that 400 mg/kg dose increased the survival time of mice significantly as compared to 100 mg/kg (*P* < 0.001) and 200 mg/kg (*P* < 0.05) treated groups. Similarly, in the aqueous fraction-treated groups, mice treated at 400 mg/kg dose survived longer than 100 mg/kg treated mice (*P* < 0.05). However, statistically significant differences were not observed in the mean survival time of mice among other doses of crude extract- and solvent fraction-treated groups (Table 2).

**Figure 1:**
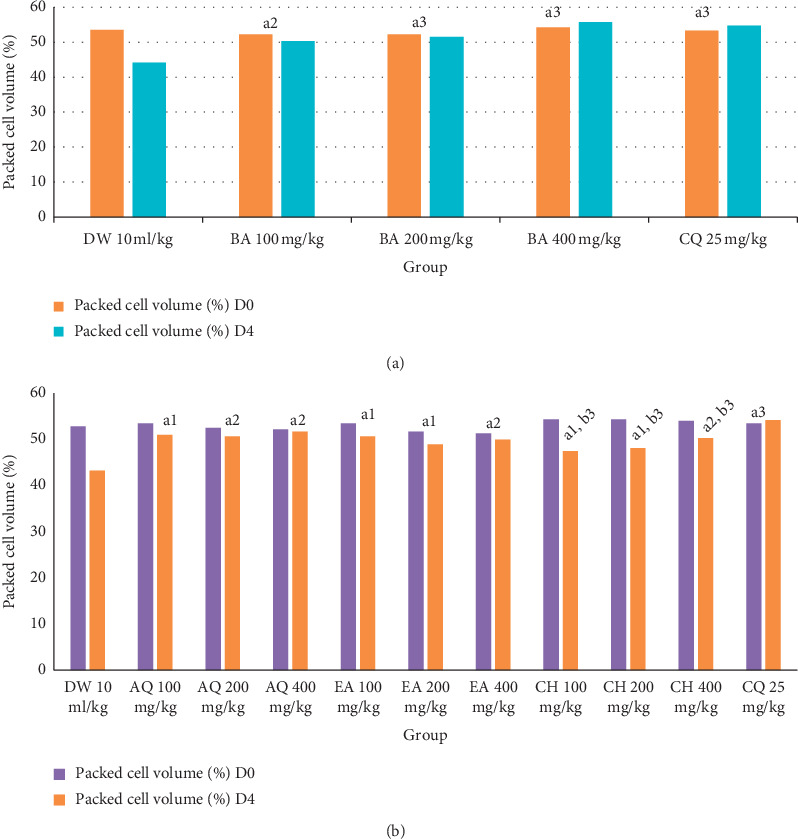
Effect of *B. abyssinica* crude extract and solvent fractions on packed cell volume of *P. berghei*-infected mice in 4-day suppressive test. Data are expressed as mean ± SEM; *n* = 6, a = compared to DW, b = compared to CQ 25 mg/kg, 1 = *P* < 0.05, 2 = *P* < 0.01, 3 = *P* < 0.001, DW = distilled water, BA = *B. abyssinica* crude extract, EA = ethyl acetate fraction, CH = Chloroform faction, AQ = aqueous fraction, CQ = chloroquine, SEM = standard error of mean, D0 = day 0, D4 = day 4.

As indicated in Figure 1, all doses of the crude extract and fractions were able to significantly (*P* < 0.05, *P* < 0.001) prevent the decrease in packed cell volume as compared to the negative control. No apparent difference was observed among the three doses of the crude extract and solvent fractions in protecting the packed cell volume of the mice. The standard drug, in comparison with all doses of the chloroform fraction (*P* < 0.05), did not show notable differences in packed cell volume protection as compared to all doses of the crude extract and other fractions.

Mice treated with all doses of the crude extract of *B. abyssinica* and solvent fractions had lost some of their body weight and rectal temperature in a non-dose-dependent manner. The crude extract and all the three fractions did not significantly protect the loss in body weight and rectal temperature as compared to the control group (Table 3). There were no significant differences among all doses of the crude extract- and solvent fraction-treated groups in the reduction of body weight and rectal temperature associated with the increasing parasitemia. A significant difference was not also noticed among crude extract- and solvent fraction- treated mice with the standard drug-treated group. However, chloroquine at all doses resulted in a significant reduction in body weight (*P* < 0.05) and rectal temperature (*P* < 0.01 and *P* < 0.001) as compared with the chloroform fraction.

### 3.4. Effect of Crude Extract and Aqueous Fraction in the Curative Test

The crude extract and aqueous fraction with a relatively highest antimalarial activity (aqueous fraction) in the four-day suppressive test were further evaluated for their effect on established parasite infection. As presented in Table 4, despite the first dose administration on the 3^rd^ day, the parasitemia level was increased on the 4^th^ day with 100 mg/kg dose of both the crude extract and aqueous fraction. Upon the second dose administration on the 4^th^ day, the parasitemia level began to decline on the 5^th^ day with all the three doses of the crude extract and the aqueous fraction and kept on decreasing across the treatment days. However, the parasitemia in the control group displayed a daily increase from day 3 to day 7. All doses of the crude extract and aqueous fractions showed a significant (*P* < 0.001) curative effect on the 7^th^ day from a thin blood film as compared to the negative control. Maximum inhibition (57.94%) was attained with a 400 mg/kg dose of crude extract. The 400 mg/kg dose of the crude extract showed significant chemosuppression as compared with the 100 mg/kg (*P* < 0.001) and 200 mg/kg (*P* < 0.05) doses of crude extract. Likewise, a comparison among aqueous fraction-treated group revealed that 400 mg/kg dose showed significant parasite suppression (*P* < 0.01) as compared to the 100 mg/kg dose.

All doses in both the crude extract and aqueous fraction, except at 100 mg/kg, significantly (*P* < 0.05–*P* < 0.001) improved survival periods of the mice as compared to the negative control. However, comparison among extract dose-treated groups did not show any apparent difference in MST of mice (Table 4).

**Figure 2:**
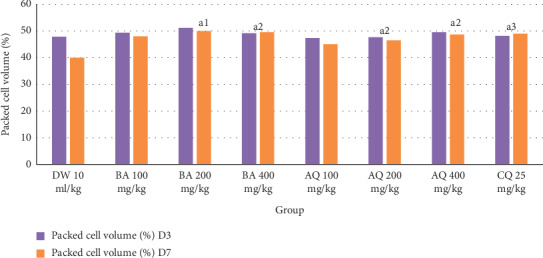
Effect of *B. abyssinica* crude extract and aqueous fraction on packed cell volume of *P. berghei-infected* mice in Rane's test. Data are expressed as mean ± SEM; *n* = 6, a = compared to DW, 1 = *P* < 0.05, 2 = *P* < 0.01, 3 = *P* < 0.001, DW = distilled water, BA = *Bersama abyssinica* crude extract, AQ = aqueous fraction, CQ = chloroquine, D3 = day 3, and D7 = day 7.

The effect of crude extract and aqueous fraction on packed cell volume is indicated in Figure 2. The decrease in PCV was significantly protected (*P* < 0.05 and *P* < 0.01) by 200 and 400 mg/kg doses of both the crude extract and aqueous fraction as compared to the negative control, respectively. However, no significant protection was observed with 100 mg/kg dose of both the crude extract and aqueous fraction as compared to the negative control. Comparison among the test groups did not demonstrate noticeable differences in preventing PCV reduction.

As shown in Table 5, the loss in body weight and rectal temperature due to malaria infection was not significantly ameliorated by all doses of the crude extract and aqueous fraction as compared to the negative control. Similarly, comparison among crude extract-treated groups and also with respect to the standard drug did not show a statistically significant difference in the prevention of body weight and rectal temperature reduction.

### 3.5. Effect of Crude Extract and Aqueous Fraction in the Prophylactic Test

The crude extract and aqueous fraction had also shown dose-dependent chemoprophylactic activities against *P. berghei*-infected mice. All doses of the crude extract and aqueous fraction significantly suppressed (*P* < 0.01 and *P* < 0.001) parasitemia as compared to the negative control. The 400 mg/kg dose of the crude extract showed significant prophylactic activity as compared with 100 mg/kg treated group. However, there were no significant differences in parasitemia suppression among the crude extract- and the aqueous fraction-treated mice. The standard drug induced a significant chemosuppression (*P* < 0.001) as compared with all doses of the crude extract and the aqueous fraction.

On the other hand, all doses of the crude extract (*P* < 0.05–*P* < 0.001) as well as 200 and 400 mg/kg doses of the aqueous fraction (*P* < 0.05 and *P* < 0.01) substantially prolonged survival time as compared to the negative control. Comparison among the crude extract dose-treated groups demonstrated that 400 mg/kg significantly prolongs (*P* < 0.05) the survival time as compared to 100 mg/kg. However, the survival times of mice treated with the aqueous fraction doses did not show significant differences (Table 6).

**Figure 3:**
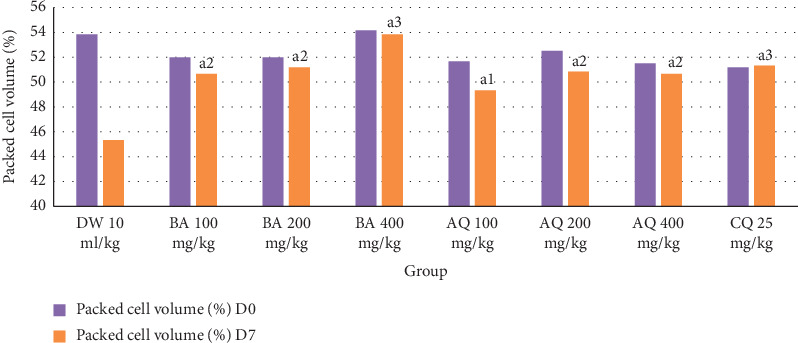
Effect of *B. abyssinica* crude extract and aqueous fraction on packed cell volume of *P. berghei*-infected mice in the prophylactic test. Data are expressed as mean ± SEM; *n* = 6, a = compared to DW, 1 = *P* < 0.05, 2 = *P* < 0.01, 3 = *P* < 0.001, DW = distilled water, BA = *Bersama abyssinica* crude extract, AQ = aqueous fraction, CQ = chloroquine, D0 = day 0, and D7 = day 7.

As depicted in Figure 3, all doses of the crude extract and the aqueous fraction were able to significantly (*P* < 0.05, *P* < 0.001) attenuate the reduction PCV in a dose-dependent manner as compared to the negative control. Comparison among crude- and aqueous fraction-treated groups including the standard drug revealed that there was no noticeable difference in the protection of packed cell volume.

Similar to the 4-day suppressive and curative tests, all doses of crude extract and aqueous fraction did not show significant protection against body weight and rectal temperature reduction as compared to the negative control. There were no considerable differences among all doses of the crude extract and aqueous fraction as well as with the standard drug in preventing body weight and rectal temperature reduction (Table 7).

## 4. Discussion

The present study explored the acute toxicity and antimalarial activity of the 80% methanolic leaf extract and solvent fractions of *B. abyssinica* against *P. berghei* infection in mice. To account a possible prodrug effect and involvement of the immune system in the eradication of infection, antimalarial studies usually employ *in vivo* models as compared to *in vitro* studies [30]. Even though primate models provide a better prediction of the evaluation of the efficacy of antimalaria in humans, rodent models are used as a first step to screen most *in vivo* antimalarial activities of tested compounds [31]. The rodent models have been also validated through the identification of several conventional antimalarial agents such as chloroquine, halofantrine, mefloquine, and artemisinin derivatives [25, 31]. It is also cost-effective in conducting preliminary pharmacological screening studies in rodent models than in primate models. The parasite *P. berghei* was used in this study since it is an appropriate parasite that is most commonly used because of its higher accessibility [31]. Due to the sensitivity and significant suppression of this parasite by chloroquine, this drug was employed as a standard [31].

The acute toxicity study revealed that the crude extract of *B. abyssinica* did not show mortality up to a dose of 2000 mg/kg. Further physical and behavioral observations also revealed no visible signs of acute toxicity with the same dose. In general, this substance is considered a good candidate for further studies since its LD50 is 20 times more than the minimum effective dose tested (100 mg/kg) which satisfies the minimum requirement [32].

The antiplasmodial properties of the crude extract and solvent fractions of the leaves of *B. abyssinica* were investigated using standard models. Accordingly, a preliminary 4-day suppressive test was conducted for the leaf extract of *B. abyssinica* to evaluate the schizonticidal activity and the percentage suppression of parasitemia of the crude extract-treated groups changed significantly from those in the negative control group. Thus, the plant is endowed with antimalarial activity and supports the previous reports of a moderate to pronounced *in vitro* antimalarial activity of leaf extracts of *B. abyssinica* [16, 17]. The methanol extract of the leaves of *Bersama engleriana*, which is found in the same genus, was reported to have highly significant *in vitro* antimalarial activity with IC50 of 2.7 *μ*g/mL [33], which further supports the result of this study. A compound is considered as active when the percentage suppression of parasitemia is 30% or more [34], which supports the findings of the current study.

Based on the preliminary suppressive effect, the crude extract was fractionated using three solvent systems (chloroform, ethyl acetate, and water) and evaluated through a 4-day suppressive test on early *Plasmodium* infection. All fractions at all doses showed significant parasitemia suppression as compared to the negative control. All the three fractions displayed significant chemosuppressive activity suggesting that the active phytochemical constituent can be extracted by all three solvents or that the antimalarial effect is produced by more than one phytochemical constituent.

However, the relative variation in chemosuppressive activity might be due to variation in secondary metabolite contents, which suggests that the more potent antimalarial phytochemical quantitatively and/or qualitatively resides within semipolar and polar solvents. From previous phytochemical screening test [20], the methanol extract showed the presence alkaloids, flavonoids, glycosides, phenols, coumarins, anthraquinones, steroids, polysterols, and triterpenes, while the ethyl acetate extract showed the presence of alkaloids, flavonoids, glycosides, phenols, coumarins, and anthraquinones but the chloroform and petroleum ether extract only showed the presence of alkaloids, flavonoids, and glycosides which all are implicated in antimalarial activity [9]. Variation in the concentration of the right subgroups of phytoconstituents responsible for the antimalarial activity might also responsible for these insignificant differences in chemosuppressive activity. The methanol, ethyl acetate, and total aqueous extracts of *B. abyssinica* were also reported to show greater microbial growth inhibition than nonpolar extracts [20, 35, 36] supporting that the biological activity of the plant lies in semipolar and polar extracts.

A fraction with highest the chemosuppression (aqueous fraction) in the 4-day suppressive test and the crude extract were further evaluated for their effect on established parasite infection using a curative test. Both the crude extract and aqueous fraction showed a significant (*P* < 0.001) curative effect at all doses as compared to the negative control with a maximum of 57.94% and 51.62% chemosuppression in the highest dose, respectively. This confirms that the plant material has effective antiplasmodial activity in the late stages of the infection. Like the standard drug, all treated groups, except 100 mg/kg dose of both the crude extract and the aqueous fraction, reduced parasitemia level after the first dose. This might be an indication of the comparative onset of defensive action of extracts and the standard drug. The delay of activity at 100 mg/kg dose of both the crude extract and the aqueous fraction might be due to the fact that the extracts at this dose had not been accumulated sufficiently to provoke considerable chemosuppression. Both the crude extract and the aqueous fraction showed dose-dependent curative antimalarial activity. The chemosuppressive effect on established infection was higher than the 4-day suppressive test, which might be due to the inhibitory effect of the extract on the generation of free radicals and hemolytic principles such as free fatty acids resulting from high parasitemia level [37].

After confirming the curative effect of the crude extract and the aqueous fraction, further evaluation was conducted to confirm their prophylactic potential, because some traditional plants which showed antiplasmodial activity in 4-day suppressive and curative tests also showed prophylactic activity against the *P. berghei* parasite [38]. In this study, the crude extract of *B. abyssinica* and the aqueous fraction had shown significant chemoprophylactic activity against residual infection at all doses as compared to the negative control with maximum parasitemia chemosuppression of 44.11% and 37.07%, respectively, at the highest dose.

The percentage suppression in the prophylactic test was found to be low as compared to 4-day suppressive and curative tests. This might have arisen from rapid hepatic clearance or metabolism of the active component responsible for antimalarial activities due to the administration of the extract initially for four days before inoculation with *P. berghei* parasite. The lower efficacy of the crude extract and fractions might be in part due to unpurified/crude nature, low selectivity, slow absorption, and poor bioavailability or other pharmacokinetic and pharmacodynamic limitations [6].

The mean survival time is another important parameter to evaluate the antimalarial activity of plant extracts [31]. In this study, the crude extract and all fractions, except the chloroform fraction, significantly prolong the mean survival time as compared to the negative control especially at higher doses in all tested models. This further supplements the evidence on the suppression of *P. berghei*, resulting in a reduced overall pathologic effect of the parasite on the study mice [39]. The mean survival time of mice treated with the standard drug was significantly prolonged (*P* < 0.001) as compared to the entire doses of the crude extract- and solvent fraction-treated groups in all models; this might be due to the fast elimination phase or less potency of the extracts. A plant material that can prolong the survival time of infected experimental animals compared to the negative control is considered as active against malaria. A principle compound that prolonged survival time beyond 12 days is regarded as active [40].

Packed cell volume reduction is one feature of malaria-infected mice. It was measured to evaluate the effectiveness of the crude extract and fractions in preventing hemolysis due to the rising parasitemia level [41]. Escalating parasitemia causes the clearance and/or destruction of infected RBCs, the clearance of uninfected RBCs, and erythropoietic suppression and dyserythropoiesis. These mechanisms have been implicated in both human and mouse malarial anemia [42]. Plants with antimalarial activity are expected to prevent a reduction in packed cell volume secondary to preventing hemolysis. Interestingly, it was noted that, in 4-day suppressive and prophylactic tests, all doses of the crude extract and solvent fractions showed significant protection in packed cell volume. In the curative test, the 200 and 400 mg/kg doses of both crude extract and the aqueous fraction also showed significant protection in packed cell volume reduction as compared with the negative control. The prevention of packed cell volume reduction might be as a result of the destructive antiplasmodial effect of the crude extract and fractions against the parasitized RBCs and the causative parasite, thereby sustaining the availability of the new RBCs produced in the bone marrow, or this might be due to the absence of saponins, which are known as phytodetergents that induce cholesterol liberation from the cell membrane and increase the permeability of the plasma membrane of the RBCs ending up with strong hemolytic effects [43, 44].

Body weight loss and rectal temperature reduction are other manifestations of rodent malaria infections that develop because of the intensity of the infection [44]. These reductions resulting from a rise in parasitemia are expected to be prevented by plants with antimalarial activity. Unfortunately, the entire doses of crude extract and solvent fractions did not prevent body weight loss and rectal temperature reduction in all tested models, even though there was significant suppression of parasitemia. This indicates the involvement of other factors for these reductions beyond malaria infection. For example, the weight loss might be due to catabolic activity on stored lipids or anorexigenic effect that may have led to decreased food intake due to the presence of appetite-suppressant metabolites in the crude extract and fractions, in addition to disturbed metabolic function and hypoglycemia related to malaria infection [39]. This appetite suppressive activity might be ascribed to flavonoids, glycosides, and phenolic compounds found in the extract [44].

On the other hand, the reduction in rectal temperature might be due to less hypothermic effect on the extract-treated mice which is supported by the use of this plant for fever management traditionally [45], in addition to the probable inability of the plant extract to ameliorate some pathological processes of malaria that cause reduction in rectal temperature or the reduction in metabolic rates that occur because of increased parasitemia [29].

Secondary metabolites have been implicated in antiplasmodial activity through different possible mechanisms including endoperoxidation by sesquiterpenes and monoterpenes [46], intercalation in DNA by anthraquinones [47], disruption of the parasite ability of detoxifying heme into nontoxic malaria pigment by alkaloids [48], blocking of protein synthesis by alkaloids and chelation with nucleic acid base pairing by flavonoids [49], immunomodulatory effects by phytosteroids and flavonoids [50], reduction of the activity of superoxide dismutase (SOD) and inhibition of the synthesis of DNA by coumarins [51], free radical scavenging effects by tannins [52], antioxidant effect by phenols like flavonoids due to their redox properties which allow them to act as reducing agents, metal chelators, and free radical quenchers [53, 54], or by any other unknown mechanisms. The antioxidative activity can inhibit haem polymerization as haem has to be oxidized before polymerization, and the unpolymerized haem is very toxic for the intraerythrocytic plasmodia [55]. The extracts could have elicited its action through any of the abovementioned mechanisms or by some other means yet to be determined. Therefore, the antiplasmodial activity observed in this plant could have resulted from a single or combined action of these metabolites.

According to Deharo et al. [56], *in vivo* antimalarial activity of plant extracts can be categorized as moderate, good, and very good if the extract showed 50% or more chemosuppression at 500, 250, and 100 mg/kg/day extract dose, respectively. Based on this rating, 80% methanolic leaf extract of *B. abyssinica* and solvent fractions have barely moderate antimalarial activity in 4-day suppressive and prophylactic tests, as none of the active doses met the threshold 50% or more inhibition, but in the curative test, both the crude extract and the aqueous fraction at 400 mg/kg dose provided a parasite suppression of >50%, exhibiting a moderate antiplasmodial activity.

## 5. Conclusion

From the results of this study, it can be concluded that the plant extract is relatively safe to mice and also showed fairly moderate antimalarial activity in early, established, and residual infections. Among fractions, the aqueous fraction showed relatively highest parasitemia suppression. The crude extract and the aqueous fraction significantly suppress parasitemia, thereby protecting the packed cell volume and prolonging the mean survival time, but failed in the prevention of rectal temperature and body weight reduction in all tested models. The overall results of this study illustrated that the leaf extracts of *B. abyssinica* have moderate antimalarial activity, and this may entail isolation and advanced safety and efficacy tests. Generally, the present pharmacological evidence provides support for the folklore claim of *B. abyssinica* leaves as an antimalarial agent and the results from previous *in vitro* studies on the antimalarial activity of the plant.

## Figures and Tables

**Table 1:** Yields of 80% methanol crude extract and solvent fractions.

Extract	Color of extract	Actual yield (g)	Percentage yield
Methanol 80% crude extract	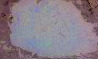	120	12
Ethyl acetate fraction (EA)	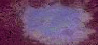	7.20	9
Chloroform fraction (CH)	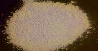	9.15	11.44
Aqueous fraction (AQ)	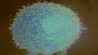	56.5	70.62

**Table 2:** Effect of *B. abyssinica* crude extract and solvent fractions on the percentage parasitemia and survival time of *P. berghei*-infected mice in the 4-day suppressive test.

Group	% parasitemia (±SEM)	% suppression	MST (days) (±SEM)
DW 10 ml/kg^*∗*^	33.57 ± 1.99	0.00	6.17 ± 0.40
BA 100 mg/kg	21.17 ± 2.25	36.94^a3,b3^	8.67 ± 0.42^b3^
BA 200 mg/kg	20.18 ± 2.23	39.89^a3,b3^	10.50 ± 0.76^a3,b3^
BA 400 mg/kg	16.95 ± 1.52	49.51^a3,b3^	13.83 ± 1.05^a3,b3,c3,d1^
CQ 25 mg/kg^*∗*^	0.00 ± 0.00	100.00^a3^	30.00 ± 0.00^a3^

DW 10 ml/kg	32.66 ± 2.59	0.00	6.33 ± 0.62
AQ 100 mg/kg	21.38 ± 3.67	34.54^a1,b3^	9.33 ± 1.30^b3^
AQ 200 mg/kg	19.67 ± 1.08	39.78^a2,b3^	10.50 ± 0.50^a1,b3^
AQ 400 mg/kg	17.08 ± 2.26	47.69^a3,b3^	13.60 ± 1.29^a3,b3,e1^
EA 100 mg/kg	20.42 ± 3.12	37.48^a2,b3^	8.83 ± 1.40^b3^
EA 200 mg/kg	20.32 ± 2.13	37.78^a2,b3^	9.17 ± 1.30^b3^
EA 400 mg/kg	18.98 ± 0.39	41.89^a2,b3^	12.00 ± 1.77^a1,b3^
CH 100 mg/kg	23.50 ± 2.13	28.05^a1b3^	8.17 ± 1.38^b3^
CH 200 mg/kg	22.48 ± 1.90	31.17^a1b3^	8.33 ± 0.95^b3^
CH 400 mg/kg	20.18 ± 2.37	38.21^a2,b3^	9.33 ± 1.67^b3^
CQ 25 mg/kg	0.00 ± 0.00	100.00^a3^	30.00 ± 0.00^a3^

Data are expressed as mean ± SEM; *n* = 6, a = compared to DW, b = compared to CQ25 mg/kg, c = compared to BA 100 mg/kg, d = compared to BA 200 mg/kg, e = compared to AQ 100 mg/kg, 1 = *P* < 0.05, 2 = *P* < 0.01, 3 = *P* < 0.001, ^*∗*^ = negative and positive controls used for crude extract in the 4-day suppressive test, DW = distilled water, BA = *B. abyssinica* crude extract, EA = ethyl acetate fraction, CH = Chloroform fraction, AQ = aqueous fraction, CQ = chloroquine, SEM = standard error of mean, and MST = mean survival time.

**Table 3:** Effect of *B. abyssinica* crude extract and solvent fractions on body weight and rectal temperature of *P. berghei*-infected mice in the 4-day suppressive test.

Group	Body weight (g) ± SEM			Rectal temperature (°c) ± SEM		
	D0	D4	% change	D0	D4	% change
DW 10 ml/kg^*∗*^	30.26 ± 0.55	28.00 ± 0.58	−7.47	36.70 ± 0.30	34.02 ± 0.22	−7.30
BA 100 mg/kg	27.42 ± 1.11	26.42 ± 0.86	−3.89	36.68 ± 0.19	35.25 ± 0.36	−3.77
BA 200 mg/kg	28.38 ± 0.51	27.51 ± 0.81	−3.14	36.35 ± 0.28	34.97 ± 0.21	−3.79
BA 400 mg/kg	28.76 ± 0.81	27.85 ± 0.95	−3.24	36.35 ± 0.28	35.08 ± 0.18	−3.48
CQ 25 mg/kg^*∗*^	27.94 ± 1.49	28.02 ± 1.36	0.29^a3^	36.13 ± 0.71	36.15 ± 0.15	0.08^a3^
DW 10 ml/kg	30.04 ± 1.78	25.39 ± 1.24	−15.48	36.18 ± 0.12	34.72 ± 0.32	−4.04
AQ 100 mg/kg	26.99 ± 0.76	24.38 ± 0.53	−8.27	36.48 ± 0.23	35.88 ± 0.22	−1.64
AQ 200 mg/kg	28.48 ± 1.02	26.64 ± 1.06	−6.46	36.52 ± 0.21	35.92 ± 0.18	−1.63
AQ 400 mg/kg	27.52 ± 0.68	26.21 ± 1.65	−5.03	36.34 ± 0.14	35.76 ± 0.81	−1.51
EA 100 mg/kg	31.73 ± 0.90	29.14 ± 1.16	−7.32	36.42 ± 0.26	35.90 ± 0.23	−1.79
EA 200 mg/kg	29.40 ± 0.76	27.32 ± 1.39	−7.07	36.37 ± 0.16	35.72 ± 0.36	−1.79
EA 400 mg/kg	32.57 ± 1.36	29.91 ± 1.41	−8.15	36.20 ± 0.23	35.80 ± 0.20	−1.10
CH 100 mg/kg	31.15 ± 0.45	28.28 ± 0.71	−9.19^b1^	36.20 ± 0.18	34.92 ± 0.31	−3.54^b3^
CH 200 mg/kg	29.43 ± 1.13	26.71 ± 0.43	−9.24^b1^	35.95 ± 0.22	34.85 ± 0.28	−3.06^b2^
CH 400 mg/kg	30.30 ± 1.34	27.63 ± 1.09	−8.81^b1^	35.82 ± 0.23	34.87 ± 0.35	−2.65^b2^
CQ 25 mg/kg	26.19 ± 1.11	26.43 ± 1.03	0.92^a3^	35.92 ± 0.28	36.57 ± 0.18	1.81^a3^

Data are expressed as mean ± SEM; *n* = 6, a = compared to distilled water, b = compared to chloroquine 25 mg/kg, 1 = *P* < 0.05, 2 = *P* < 0.01, 3 = *P* < 0.001, ^*∗*^ = negative and positive controls used for crude extract in the 4 day suppressive test, DW = distilled water, BA = *B. abyssinica* crude extract, EA = ethyl acetate fraction, CH = chloroform fraction AQ = aqueous fraction, CQ = chloroquine, SEM = standard error of mean, D0 = day 0, and D4 = day 4.

**Table 4:** Effect of *B. abyssinica* crude extract and aqueous fraction on percentage parasitemia, and survival time of *P. berghei*-infected mice in Rane's test.

Group	% parasitemia (±SEM)					% suppression	MST (days)
	D3	D4	D5	D6	D7		
DW 10 ml/kg	21.63 ± 0.33	23.72 ± 0.68	25.27 ± 1.61	27.12 ± 0.78	29.68 ± 0.82	0.00	8.17 ± 0.48
BA 100 mg/kg	24.01 ± 0.48	24.30 ± 0.70	22.67 ± 0.92^b3^	21.57 ± 1.64^b3^	19.95 ± 1.42^a3,b3^	34.37^a3,b3^	13.29 ± 1.41^b3^
BA 200 mg/kg	24.92 ± 0.83	24.07 ± 1.00	23.42 ± 1.36^b3^	22.05 ± 2.91^b3^	16.86 ± 1.08^a3,b3^	43.19^a3,b3^	14.50 ± 1.82^a2,b3^
BA 400 mg/kg	25.05 ± 0.75	24.13 ± 0.76	21.87 ± 0.59^b3^	19.75 ± 0.99^b3^	12.48 ± 0.77^a3,b3,c3,d1^	57.94^a3,b3,c3,d1^	16.67 ± 0.49^a3,b3^
AQ 100 mg/kg	24.88 ± 0.62	26.06 ± 0.65^b3^	24.78 ± 0.90^b3^	22.12 ± 0.89^b3^	19.87 ± 0.56^a3,b3^	31.40^a3,b3^	11.60 ± 1.44^b3^
AQ 200 mg/kg	24.73 ± 0.97	24.67 ± 0.97^b1^	22.12 ± 0.64^b3^	21.60 ± 1.34^b3^	17.58 ± 1.44^a3,b3^	40.77^a3,b3^	13.83 ± 1.60^a1,b3^
AQ 400 mg/kg	25.18 ± 0.80	24.03 ± 0.91^b1^	22.87 ± 1.22^b3^	20.92 ± 1.59^b3^	14.36 ± 1.23^a3,b3,e2^	51.62^a3,b3,e2^	15.17 ± 1.87^a2,b3^
CQ 25 mg/kg	22.68 ± 0.78	20.47 ± 1.07^a1^	13.72 ± 1.51^a3^	5.28 ± 2.42^a3^	0.00 ± 0.00^a3^	100.00^a3^	30.00 ± 0.00^a3^

Data are expressed as mean ± SEM; *n* = 6, a = compared to DW, b = compared to CQ 25 mg/kg, c = compared to BA 100 mg/kg, d = compared to BA 200 mg/kg, e = compared to AQ 100 mg/kg, 1 = *P* < 0.05, 2 = *P* < 0.01, 3 = *P* < 0.001, DW = distilled water, BA = *Bersama abyssinica* crude extract, AQ = aqueous fraction, CQ = chloroquine, SEM = standard error of mean, MST = mean survival time, D3 = day 3, D4 = day 4, D5 = day 5, D6 = day 6, and D7 = day 7.

**Table 5:** Effect of *B. abyssinica* crude extract and aqueous fraction on body weight and rectal temperature of *P. berghei-*infected mice in Rane's test.

Group	Body weight (g) ± SEM			Rectal temperature (°C) ± SEM		
	D3	D7	% change	D3	D7	% change
DW 10 ml/kg	24.84 ± 1.86	22.10 ± 2.12	−11.03	35.17 ± 0.18	33.53 ± 0.34	−4.64
BA 100 mg/kg	25.90 ± 0.47	23.01 ± 0.79	−10.45	35.37 ± 0.37	34.23 ± 0.21	−3.17
BA 200 mg/kg	27.84 ± 0.64	24.90 ± 0.85	−10.56	35.65 ± 0.37	34.47 ± 0.30	−3.28
BA 400 mg/kg	28.74 ± 0.95	26.42 ± 0.84	−8.07	36.00 ± 0.28	34.90 ± 0.32	−3.04
AQ 100 mg/kg	24.55 ± 1.86	22.54 ± 0.64	−9.10	35.73 ± 0.42	34.26 ± 0.30	−4.07
AQ 200 mg/kg	27.28 ± 0.94	24.76 ± 0.66	−9.24	35.35 ± 0.25	34.00 ± 0.40	−3.79
AQ 400 mg/kg	27.18 ± 0.33	25.44 ± 1.21	−6.40	35.02 ± 0.30	33.93 ± 0.53	−3.07
CQ 25 mg/kg	28.37 ± 1.16	28.17 ± 1.04	−0.60^a1^	35.65 ± 0.46	36.36 ± 0.26	2.07^a1^

Data are expressed as mean ± SEM; *n* = 6, a = compared to DW, 1 = *P* < 0.05, 2 = *P* < 0.01, 3 = *P* < 0.001, DW = distilled water, BA = *Bersama abyssinica* crude extract, AQ = aqueous fraction, CQ = chloroquine, SEM = standard error of the mean, D3 = day 3, and D7 = day 7.

**Table 6:** Effect of *B. abyssinica* crude extract and aqueous fraction on percentage parasitemia and survival time of *P. berghei*-infected mice in the prophylactic test.

Treatment	% parasitemia (±SEM)	% suppression	MST (days) (±SEM)
DW 10 ml/kg	30.30 ± 1.30	0.00	7.83 ± 0.87
BA 100 mg/kg	22.10 ± 0.93	27.06^a3,b3^	11.33 ± 0.71^a1,b3^
BA 200 mg/kg	19.65 ± 1.22	35.15^a3,b3^	12.17 ± 0.95^a2,b3^
BA 400 mg/kg	16.93 ± 1.00	44.11^a3,b3,c2^	14.67 ± 0.88^a3,b3,c1^
AQ 100 mg/kg	22.98 ± 1.06	24.16^a2,b3^	9.83 ± 1.45^b3^
AQ 200 mg/kg	21.20 ± 0.75	30.03^a3,b3^	11.33 ± 1.12^a1,b3^
AQ 400 mg/kg	19.07 ± 2.10	37.07^a3,b3^	13.50 ± 1.65^a2,b3^
CQ 25 mg/kg	0.30 ± 0.18	99.01^a3^	29.60 ± 0.40^a3^

Data are expressed as mean ± SEM; *n* = 6, a = compared to DW, b = compared to chloroquine 25 mg/kg, c = compared to BA 100 mg/kg, 1 = *P* < 0.05, 2 = *P* < 0.01, 3 = *P* < 0.001, DW = distilled water, BA = *Bersama abyssinica* crude extract, AQ = aqueous fraction, CQ = chloroquine, SEM = standard error of the mean, MST = mean survival time.

**Table 7:** Effect of *B. abyssinica* crude extract and aqueous fraction on body weight and rectal temperature of *P. berghei*-infected mice in the prophylactic test.

Group	Wt (g) ± SEM			T (°c) ± SEM		
	D0	D7	% change	D0	D7	% change
DW 10 ml/kg	24.17 ± 1.97	21.41 ± 0.91	−11.42	36.57 ± 0.22	34.85 ± 0.28	−4.70
BA 100 mg/kg	24.12 ± 0.36	22.10 ± 0.62	−8.43	37.17 ± 0.14	36.18 ± 0.24	−2.64
BA 200 mg/kg	26.39 ± 0.73	24.16 ± 0.68	−8.45	36.88 ± 0.25	36.03 ± 0.15	−2.28
BA 400 mg/kg	25.64 ± 0.34	23.58 ± 0.40	−8.03	36.78 ± 0.12	35.97 ± 0.20	−2.22
AQ 100 mg/kg	23.36 ± 0.58	21.39 ± 0.49	−8.46	36.65 ± 0.14	35.67 ± 0.30	−2.68
AQ 200 mg/kg	24.07 ± 0.64	22.09 ± 0.78	−8.24	37.17 ± 0.24	36.25 ± 0.16	−2.45
AQ 400 mg/kg	25.67 ± 1.29	23.48 ± 1.17	−8.30	37.20 ± 0.21	36.37 ± 0.32	−2.23
CQ 25 mg/kg	23.26 ± 1.05	23.85 ± 1.16	2.54^a1^	36.52 ± 0.22	36.72 ± 0.17	0.55^a2^

Data are expressed as mean ± SEM; *n* = 6, a = compared to DW, 1 = *P* < 0.05, 2 = *P* < 0.011, 3 = *P* < 0.001, DW = distilled water, BA = *Bersama abyssinica* crude extract, AQ = aqueous fraction, CQ = chloroquine, SEM = standard error of mean, MST = mean survival time, D0 = day 0, D7 = day 7, T = temperature, and Wt = body weight.

## Data Availability

The datasets used and/or analyzed during the current study are available from the corresponding author upon reasonable request.

## Abbreviations

PCV: Packed cell volume
RBC: Red blood cell
SEM: Standard error of the mean
OECD: Organization for Economic Cooperation
ILAR: Institute for Laboratory Animal Research.
